# The Effect of the Position Determination Error for Flexible Linear Array Elements on the Tomogram Focusing

**DOI:** 10.3390/s23104757

**Published:** 2023-05-15

**Authors:** Dmitry A. Sednev, Alexey I. Soldatov, Andrey A. Soldatov, Maria A. Kostina, Daria A. Koneva

**Affiliations:** School of Non-Destructive Testing, National Research Tomsk Polytechnic University, 30 Lenin Avenue, 634050 Tomsk, Russia

**Keywords:** curved surface, flexible acoustic array, focusing, Strehl ratio, total focusing method, tomogram, wavelength

## Abstract

In the article, the study of the quality of tomogram focusing during the inspection of objects with curved surfaces by flexible acoustic array was described. The main goal of the study was theoretically and experimentally define the acceptable deviation limits of the elements’ coordinates values. The tomogram reconstruction was performed by the total focusing method. The Strehl ratio was chosen as a criterion for assessing the quality of tomogram focusing. The ultrasonic inspection procedure were simulated and validated experimentally by means of convex and concave curved arrays. In the study, it was proven that the elements coordinates of the flexible acoustic array were determined with an error of no more than 0.18λ and the tomogram image was obtained in sharp focus.

## 1. Introduction

In recent years, the multi-element acoustic arrays found a broad range of applications for inspection of industrial components [1,2,3,4,5,6,7,8,9]. Among the advantages in comparison with the single-element transducers, it is possible to mention the following:

- Much faster inspection process;
- Easiness in reading ultrasonic image;
- Imperfection may be immediately detected and evaluated;
- Several types of inspection may be realised using the same equipment;
- Possibility to reconstruct 2D and 3D images [10,11];
- Possibility to gather inspection data for large inspection zone from a single transducer position.

As a result, an inspector has a possibility to detect imperfections, but also to evaluate their quantitative parameters and relative location. In order to obtain a clear image of the object structure, it is required to focus the acoustic energy in each point of the inspection zone. Focusing may be realised by setting delay laws for each element of the acoustic array in order to direct the maximum acoustic energy to the certain point of an inspected object structure [1]. Additionally, focus may be achieved synthetically by shifting of A-scans during the post-processing stage [7,8,9,10,11]. There are several methods of reconstruction of the acoustic signals obtained from the acoustic array used in ultrasonic non-destructive testing. The most widely spread among them is the common source method (CSM), synthetic aperture focusing technique (SAFT) and total focusing method (TFM).

The TFM is considered as the most informative reconstruction method and is named “the gold standard” in advanced non-destructive testing. The delay times from the transmitter to the certain point in the inspection zone and, from this point to the receiver, need to be determined in order to reconstruct the tomogram. When the delay law is known, it is possible to determine the amplitude of echo signal and calculate the amplitude of each pixel of the tomogram according to Kirchhoff migration [12]. This approach is used both for objects with a flat surface and a curved one [13,14,15,16,17,18,19]. The range of curve shaped objects is rather wide, such as crankshafts and camshafts of internal combustion engines, blades of gas turbine engines, blades of water and steam turbines, bearings, wheels and axles of railway cars, etc. The necessity of non-destructive testing of curve shaped units is noted in [20,21,22,23,24]. The most widely spread unit with a curved surface is the crankshaft of an internal combustion engine. The high cost, complexity of manufacturing and high operating loads determine the high requirements for the quality of crankshaft manufacturing. 

Quality assurance procedure by means of flexible acoustic arrays is one of the advanced inspection types for objects with a curved surface. The application of flexible acoustic arrays in non-destructive testing leads to need of determination the array elements coordinates to obtain the focused reconstructed acoustic imaging of the inspection zone by TFM. The pixel intensity in TFM post-processing is calculated by the equation:

(1) $$I\left(x,y\right)=\sum_{j}^{n}\sum_{i}^{n}A_{i,j}\left(t-t_{i,j}\right),$$

where $I\left(x,y\right)$—pixel intensity with coordinates $\left(x,y\right)$, $t_{i,j}$—delay time for acoustic signal emitted from a transmitting array element *i* to a receiving array element *j* through the point with coordinates $\left(x,y\right)$, *n*—the number of elements in the array.

The delay time $t_{i,j}$ is determined by (2):

(2) $$t_{i,j}=\frac{\sqrt{{\left(x_{i}-x\right)}^{2}+{\left(y_{i}-y\right)}^{2}}+\sqrt{{\left(x_{j}-x\right)}^{2}+{\left(y_{j}-y\right)}^{2}}}{c}$$

where $\left(x,y\right)$—pixel coordinates, $\left(x_{i},y_{i}\right)$—coordinates of the transmitting array element, $\left(x_{j},y_{j}\right)$—coordinates of the receiving array element, c—elastic wave propagation velocity in the medium.

The most precise focus will be obtained when all values $A_{i,j}\left(t-t_{i,j}\right)$ are coherent; thus, the amplitude of the reflector on the tomogram is maximum.

The coordinates of the elements of the flexible acoustic array with variable configuration are a priori unknown and determined with an error described by $\left(x_{i}+∆x_{i},y_{i}+∆y_{i}\right)$ and $\left(x_{j}+∆x_{j},y_{j}+∆y_{j}\right)$. This leads to incorrect determination of a delay law:

(3) $$\tilde{t_{i,j}}=\frac{\sqrt{{\left(x_{i}+∆x_{i}-x\right)}^{2}+{\left(y_{i}+∆y_{i}-y\right)}^{2}}+\sqrt{{\left(x_{j}+∆x_{j}-x\right)}^{2}+{\left(y_{j}+∆y_{j}-y\right)}^{2}}}{c},$$

where $∆x_{i}$—error in determining the coordinate $x_{i}$ of the transmitting array element, $∆y_{i}$—error in determining the coordinate $y_{i}$ of the transmitting array element, $∆x_{j}$—error in determining the coordinate $x_{j}$ of the receiving array element, $∆y_{j}$—error in determining the coordinate $y_{j}$ of the receiving array element.

Errors in a delay law determination lead to violation of coherent summation and, as a consequence, to incorrect determination of the amplitude of each pixel in the inspection zone:

(4) $$\tilde{I}\left(x,y\right)=\sum_{j}^{n}\sum_{i}^{n}A_{i,j}\left(t-\tilde{t_{i,j}}\right),$$

Phased array ultrasonic testing technique permits beam steering in an inspection zone. The ultrasonic beam focusing to a certain point is performed by setting a delay law for the acoustic array, so that acoustic waves from all elements of the array arrive at the focusing point simultaneously. Zero delay time is set to element that is nearest to a focus point. The delay law for other elements is calculated in accordance with the equation:

(5) $${∆t_{i}=t}_{i}-t_{0}=\frac{\sqrt{{\left(x_{i}-x\right)}^{2}+{\left(y_{i}-y\right)}^{2}}}{c}-\frac{\sqrt{{\left(x_{0}-x\right)}^{2}+{\left(y_{0}-y\right)}^{2}}}{c},$$

where $t_{0}$—time of flight of an acoustic wave from a nearest element to a focus point, $t_{i}$—time of flight of an acoustic wave from the *i* element of the acoustic array to a focus point, $\left(x,y\right)$—coordinates of a focus point, $\left(x_{i},y_{i}\right)$—coordinates of the transmitting array element.

The incorrect determination of the array elements coordinates will cause errors in defining of the delay law that could be described by the following equation:

(6) $${\tilde{∆t_{i}}=t}_{i}-t_{0}=\frac{\sqrt{{\left(x_{i}+∆x_{i}-x\right)}^{2}+{\left(y_{i}+∆y_{i}-y\right)}^{2}}}{c}-\frac{\sqrt{{\left(x_{0}+∆x_{0}-x\right)}^{2}+{\left(y_{0}+∆y_{0}-y\right)}^{2}}}{c},$$

where $∆x_{i}$—error in determining the coordinate $x_{i}$ of the transmitting array element, $∆y_{i}$—error in determining the coordinate $y_{i}$ of the transmitting array element,$∆x_{0}$—error in determining the coordinate $x_{0}$ of the transmitting array element nearest of the focus point, $∆y_{0}$—error in determining the coordinate $y_{0}$ of the transmitting array element nearest of the focus point.

**Figure 1:**
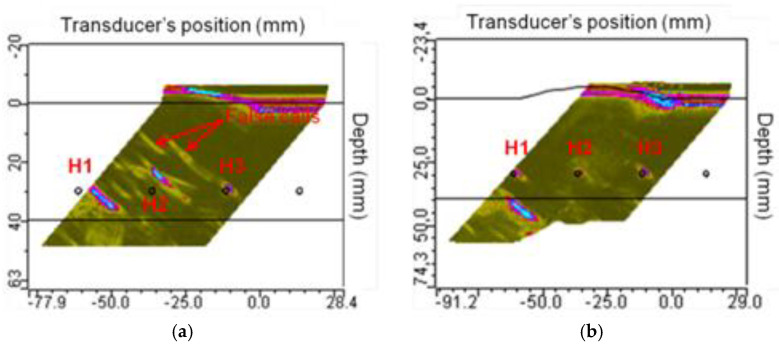
The contribution of the surface profile in flaw detection: (**a**) delay laws computed for a planar surface; (**b**) delay laws are adapted to the curved geometry [13].

The results of incorrect determination of a delay law are shown in Figure 1 [13]. The different approach to delay law computation for inspection of the curved object led to the appearance of false calls of artefacts when the delay laws are not adopted to a geometry (Figure 1a). However, the shift in a transducer element’s coordinates will allow to obtain precise data about the inner structure (Figure 1b).

In [14], the authors presented a delay law for the 64 elements acoustic array, calculated by two different methods: a built-in algorithm and theoretical values calculated by CIVA modelling software tool. The maximum difference was 0.15 µs, which corresponds to a distance of 0.9 mm in steel. An incorrect determination of the delay law leads to a change the focal spot dimensions in more than two times and a change in the amplitude of the ultrasonic signal in the range from −2.5 dB to +2.0 dB [14]. In addition, the signal-to-noise ratio decreases and information about small reflectors may be lost or underestimated, artifacts may appear on the tomogram, as well as increasing the size of the reflectors and incorrect determining of its coordinates [25,26,27,28,29]. 

Therefore, it is important to determine the acceptable deviation of the elements’ coordinates values. The main goal of the study is theoretically, by calculation and simulation, define the acceptable deviation limits and validate it by experiment.

One of the options to obtain precise elements’ coordinates is the application of the flexible acoustic array with fiber-optic sensor, which measures the positions of acoustic array elements [15]. The fiber-optic systems capable to vary the optical signal characteristics. A change in the values of cable curvature will lead to the various scattering types of optical signal: Rayleigh, Brillouin and Roman. It is possible to use the classical theory of optical interferometry to analyse the deformation of the fiber-optic cable. However, the limitations related to fiber-optic sensors do not allow inspection of objects with the curvature radius less than 125 mm. The studies performed by the authors [15] revealed the position determination error of the acoustic array by means of the fiber-optic system. However, the dependence between errors in coordinates and the imperfection of reconstruction result was not studied.

Another approach to use the “smart flexible array” technique is combination of flexible acoustic array and integrated profilometer to measure local curvatures of the surface and to calculate the delay law in real time mode. Two-dimensional-flexible acoustic arrays are manufactured by IMASONIC [16]. Although it is worth mentioning that the integrated profilometer increases the acoustic sensors dimensions and it becomes the limitation for practical application under the conditions of limited access to an inspected object. 

The effect of an irregular surface on the inspection results was studied by S. Mahaut, J. Porre, P. Calmon, S. Chatillion and O. Roy [17] both for contact and immersion acoustic sensors. These authors discussed an application the same delay law for isotropic and anisotropic media. In case of acoustic signal propagation through anisotropic structure, signal will change the refraction angle as well as have a deeper focus point in comparison with an isotropic structure. The acoustic signals aberration was observed during inspection of an irregular surface, because the delay law was calculated in relation to the flat interface. This caused the deflection of acoustic signals and the best focusing was observed in the nearest positions to the minimal curvature radius of an object surface.

The surface profile evaluation using several ultrasonic techniques was performed by S. Robert with co-authors [18]. These authors evaluated the surface profile using pulse- echo, pitch-catch, TFM and surface adaptive ultrasounds (SAUL) inspection modes. The authors demonstrated that the error of the profile obtained by pulse-echo mode was λ/4, while using the pitch-catch mode and TFM, it was λ/8, while the SAUL error was λ/2. The authors discussed the reconstructed acoustic image of the inspection zone with curved surface and defects within the inspection zone. However, the evaluation of focusing quality of the reconstructed image was not performed. 

## 2. Evaluation Criteria

The curved acoustic array model is used to evaluate the effect of the position determination error for the acoustic array elements on the quality of the tomogram focusing. The Strehl ratio [30], Rayleigh criterion [31] or Marechal [32] are applied for evaluating the image focusing quality. The degradation in the focusing quality led to appearance of aberrations, which are revealed in the decreasing of the amplitude of the central maximum of the function of the scattering point (Airy disk). The Strehl ratio is considered as acceptable when the decreasing amplitude of the central maximum (coordinate X = 0) of the point spread function (PSF) does not exceed 20%:

(7) $$St=\frac{h_{exp}}{h_{th}},$$

where *h_exp_*—current experimental value of the amplitude of the central maximum, *h_th_*—maximum theoretical value of the amplitude of the central maximum.

**Figure 2:**
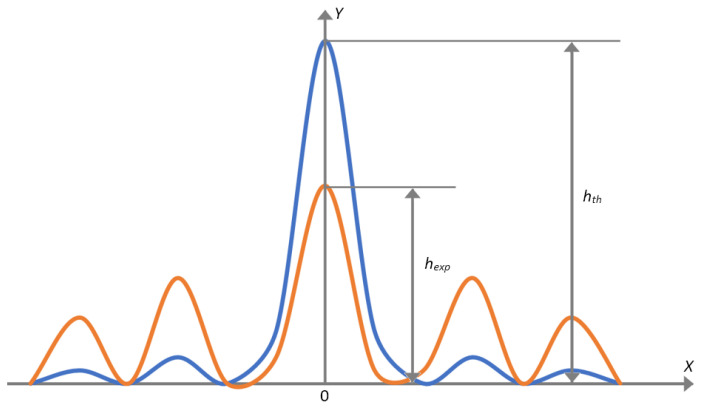
The amplitude distribution of PSF, where the blue line is the precise focusing and the red line is for focusing degradation.

The example of changing of the amplitude of the central maximum of PSF under the degradation in the image focusing quality is presented in Figure 2.

The Rayleigh criterion is in the following: the image is considered to be focused in case the value of wave aberration does not exceed one fourth of the wavelength. At the same time, the Strehl ratio is more than or equal to 0.8 [30].

In case the mean square value of the wave aberration, called the Marechal approximation, does not exceed 5%, the Strehl ratio is more than or equal to 0.8. The Marechal approximation is valid for residual aberrations for any types of aberrations of small values [33]. 

In non-destructive testing, the point reflector is used to determine the characteristics of an equipment. That is why it is the most convenient way to use the Strehl ratio for evaluation of the degradation of the tomogram focusing quality caused by the error of position determination of acoustic array elements. There are evaluated changes in amplitude of the central maximum of the point reflector, the acoustic image of which is presented by PSF.

## 3. Simulation

The simulation was conducted for 16, 32 and 64 elements acoustic arrays with convex and concave shapes and a radius of curvature equal to 30 and 60 mm. The selected values of curvature corresponded to the dimensions of the crankshaft neck and crank bearing of the crankshafts of trucks. For example, for KAMAZ trucks, the diameter of the crankshaft neck was 95 mm and the diameter of the crank bearing was 80 mm. The SCANIA had 119 mm and 108 mm, respectively. In accordance with these dimensions, the reflector location ranges of 5, 10, 15, 24, 35 and 50 mm were selected. The simulation was performed for following arrays frequencies 1.5, 3 and 5 MHz, which corresponds to acoustic wavelengths of 1.0, 0.5 and 0.3 mm, accordingly. 

**Figure 3:**
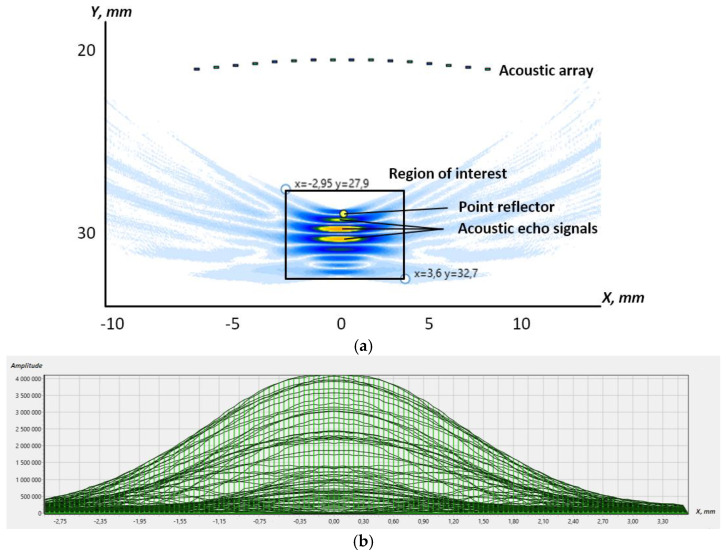
The reconstruction of the RoI with the point reflector: (**a**) the tomogram; (**b**) the point reflector’s amplitude distribution.

Figure 3 demonstrates the results of the reconstruction of the inspection zone with the point reflector. The simulation parameters were the following: the number of the array elements was 16, the curvature radius was 30 mm, the distance between the array centre and the point reflector was 5 mm, the pitch was 1 mm and the frequency was 1.5 MHz. At the top of Figure 3a, dots depict the configuration of the acoustic array. The acoustic image of the point reflector is shown at the bottom. The inspection zone was reconstructed by TFM. The simulation software allows users to set region of interest (RoI) where the pixel amplitude can be measured and displayed. The Hilbert transform was performed for the RoI. That allows one to determine the maximum value of the amplitude *h_th_* for the point reflector. Figure 3b shows the amplitude distribution after performing the Hilbert transform for the RoI shown in Figure 3a. 

The methodology for evaluation of the acceptable determination error is the following:

1. The initial data are generated with using the precise coordinates of the acoustic array;
2. The reconstruction of an acoustic image was performed with shift of the array elements coordinates by the value of 0.05λ;
3. The even elements of the acoustic array being shifted by +0.05λ along the “Y” axis;
4. The odd elements of the acoustic array being shifted by −0.05λ along the “Y” axis;
5. Thus, the maximum phase shift between A-scans of even and odd elements will be achieved;
6. The maximum value of the function $h_{0}\left(0\right)$ was determined for each shift.

**Figure 4:**
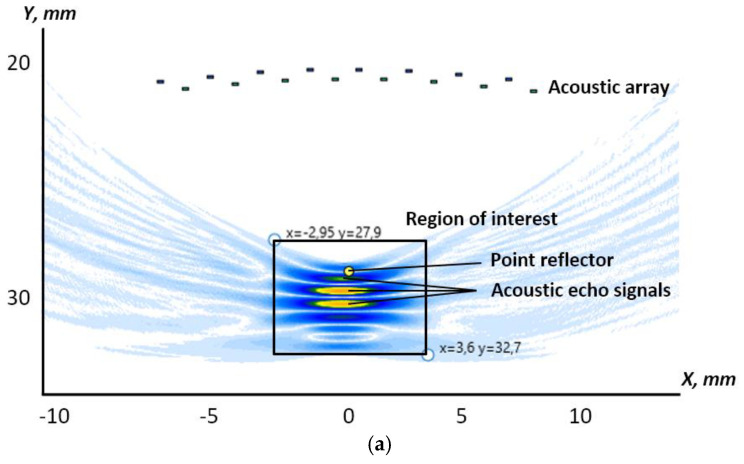

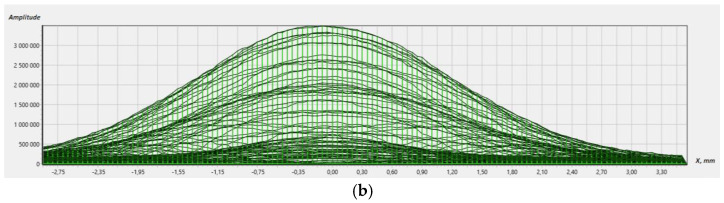
The reconstruction of the RoI with the shifted coordinates: (**a**) the tomogram; (**b**) the point reflector’s amplitude distribution.

In Figure 4, the reconstruction for the configuration of the array elements with applied shift is shown.

**Figure 5:**
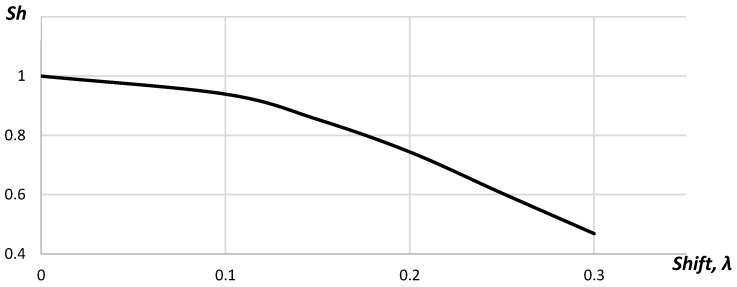
The dependence of the Strehl ratio on the shift.

In Figure 5, the dependence of the Strehl ratio (*Sh*), calculated with Equation (7), from the shift of the acoustic array elements is given.

**Figure 6:**
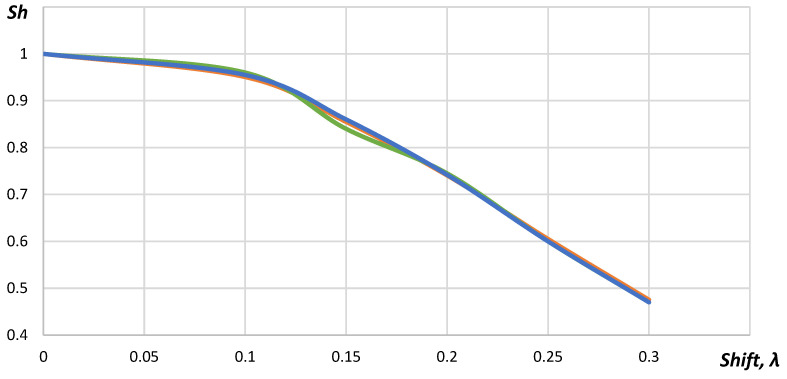
The dependence of the Strehl ratio on the shift: the blue line—the point reflector distance from the acoustic array equals 5 mm; the green line—10 mm; the orange line—15 mm.

In Figure 5, it is shown that the Strehl Ratio decreased gradually from 1.00 to 0.47 with the increase in the shift. The studies performed for 32 elements acoustic array showed that the requirements to the position determination accuracy did not change. The Strehl ratio was below 0.8 for the acoustic array elements shifting up to 0.18λ for the cases when the point reflector was placed in the distance 5, 10 and 15 mm from the acoustic array. Figure 6 represents the Strehl ratio dependences on the shift for different positions of the point reflector.

As seen in Figure 6, the dependence between the Strehl ratio and the shift did not correlate with the distance to the point reflector. Increasing number of elements in the acoustic array up to 64 did not influence the requirements to the position determination accuracy.

The dependence between the Strehl ratio and the shift for 64, 32 and 16 elements acoustic arrays corresponded to each other. The changes of the acoustic array curvature did not influence the dependence between the Strehl ratio and the shift. Thus, the requirements to the accuracy in the position determination of the acoustic array elements did not change, also. 

The decreasing of the wavelength value two times (λ = 0.5 mm) led to the reduction in the acceptable coordinates shift in two times, respectively. The value of the acceptable shift for the elements of the concave acoustic array with a radius of 60 mm is given in Table 1. The values were calculated for the distance between the array and the point reflector was 5, 10, 15, 24, 35 and 50 mm.

**Figure 7:**
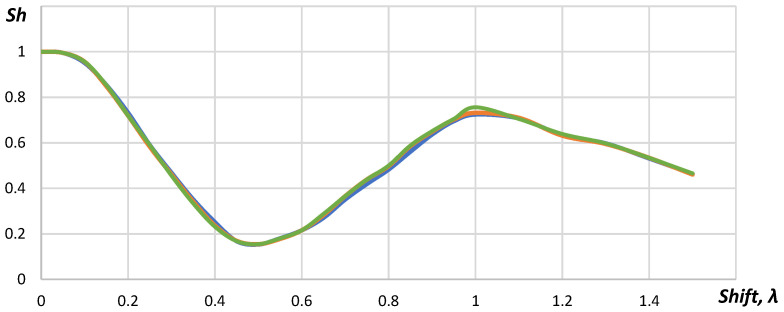
The dependence between the Strehl ratio and the shift: the blue line—the point reflector distance from the acoustic array equals to 24 mm; the green line—35 mm; the orange line—50 mm.

In Figure 7, it is shown that dependences of the Strehl ratio for distances were 24, 35 and 50 mm, which corresponded to each other. The minimum value of the Strehl ratio was reached with the shift by 0.5λ. In this case, the phase shift between A-scans of even and odd elements reached 180 degrees. When the coordinates of the elements were shifted by λ, the phase shift reached 360 degrees and another maximum appeared. However, the Strehl ratio for this case did not exceed 0.75. 

## 4. Experimental Validation

**Figure 8:**
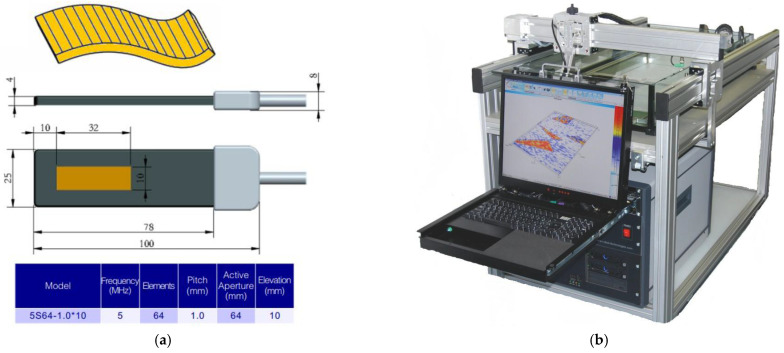
The equipment: (**a**) the multi-element flexible acoustic array 5S64-1.0*10 [34]; (**b**) the multi-channel ultrasonic inspection system IDEAL-SYSTEM [35].

To confirm the simulation results, an experimental validation of the tomogram focusing quality was performed. The experiment was designed to use the following equipment: the 64 element linear flexible acoustic array 5S64-1.0×10 produced by DOPPLER (Guangzhou Doppler Electronic Technologies Co., Ltd., Guangzhou, China) (Figure 8a), the multichannel ultrasonic inspection system IDEAL-SYSTEM produced by I-DEAL Technologies (Saarbrücken, Germany) (Figure 8b).

**Figure 9:**
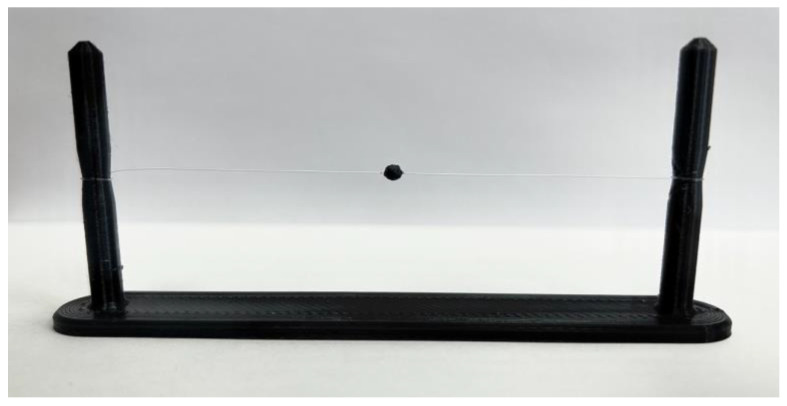
The experimental sample for the validation procedure.

Experimental sample was prepared in order to perform validation procedure. The sample consisted of U-shaped plate made by means of additive manufacturing and, between two side pins, a wire with the diameter of 0.05 mm was strained. The wire’s diameter was much smaller than the acoustic wavelength of the DOPPLER array which equalled roughly 0.3 mm. In this condition, the wire was transparent for an acoustic signal and suitable for positioning of the point reflector. The experimental sample is presented in Figure 9.

**Figure 10:**
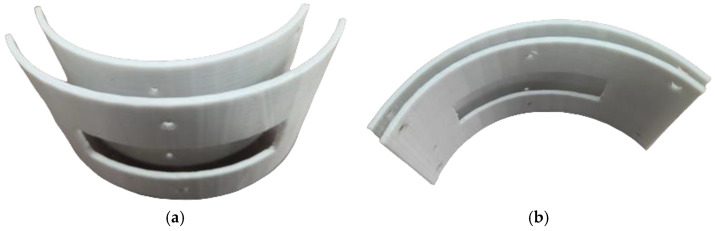
The templates for the flexible acoustic array: (**a**) the convex; (**b**) the concave.

In order to obtain the necessary curvature for the flexible acoustic array, there were prepared two templates. The first template shaped the array as convex with the curvature radius of 60 mm (Figure 10a), while the second template gave to the array a concave shape with the same radius of 60 mm (Figure 10b). The radius value was limited by technical capabilities of the acoustic array. According to the technical specification, the minimum curvature radius for the flexible acoustic array 5S64-1.0*10 was equal to 60 mm.

**Figure 11:**
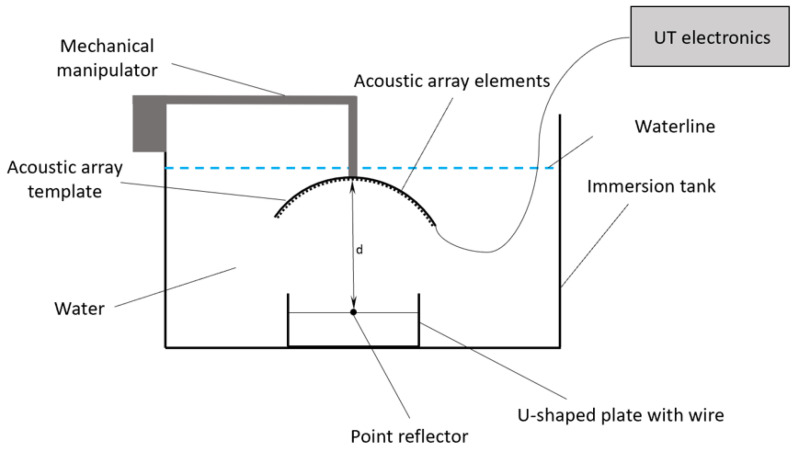
The experiment design scheme.

The principal design of the experiment is shown in Figure 11. The flexible acoustic array with 64 elements was placed into template of the concave shape and was mounted on a mechanical manipulator of ultrasonic inspection system. The array was connected to IDEAL-SYSTEM ultrasonic electronics with 16 channels and multiplexing possibility. The flexible acoustic array was located in an immersion tank filled with water as well as a U-shaped plate with a pointed reflector mounted by a thin wire. The distance between the central element of acoustic array and the point reflector was named “d” and was equal to 24, 35, 42 and 50 mm in different series of experiments. 

**Figure 12:**
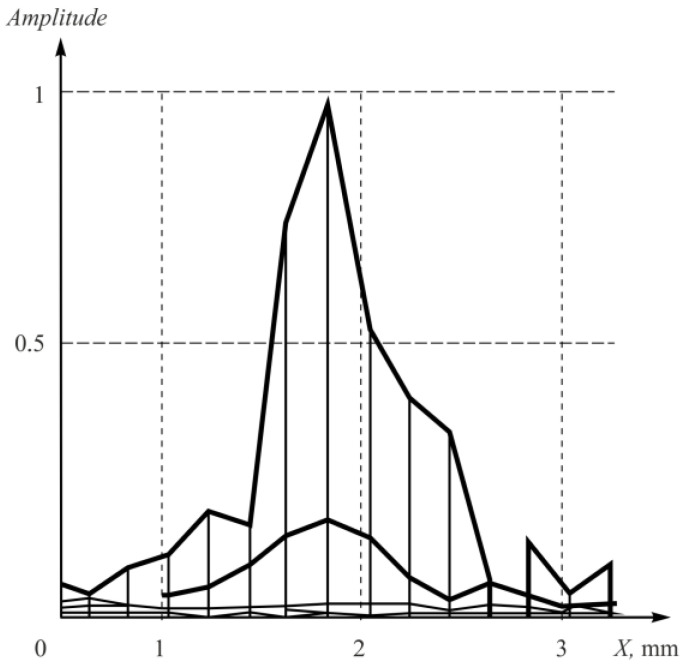
The experimental amplitude distribution of the PSF for the point reflector.

The experimental amplitude distribution of the PSF was calculated by obtaining the raw experimental data and following tomogram reconstruction using the array elements’ coordinates. Then, the transverse projection of the point reflector’s amplitude was determined and plotted (Figure 12).

Then, the coordinate shift of even elements increased while the coordinates of odd elements decreased by the same value. After that, the reconstruction of tomogram of the inspection zone was conducted. The transverse projection of the RoI with the point reflector was plotted. The Hilbert transform was applied to the plotted projection in order to obtain the envelope curve. Then, the reconstructed amplitudes of the point reflector were measured. The results of the measurements are given in Table 2.

**Figure 13:**
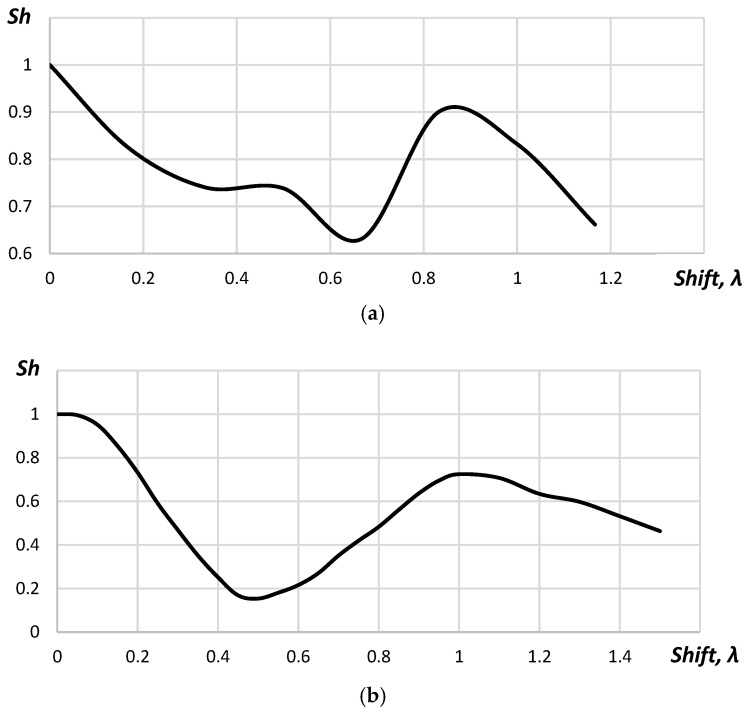
The dependence between the Strehl ratio and the shift: (**a**) experimental validation, (**b**) simulation.

The dependence between the Strehl ratio and the shift of the flexible array elements is provided in Figure 13.

**Figure 14:**
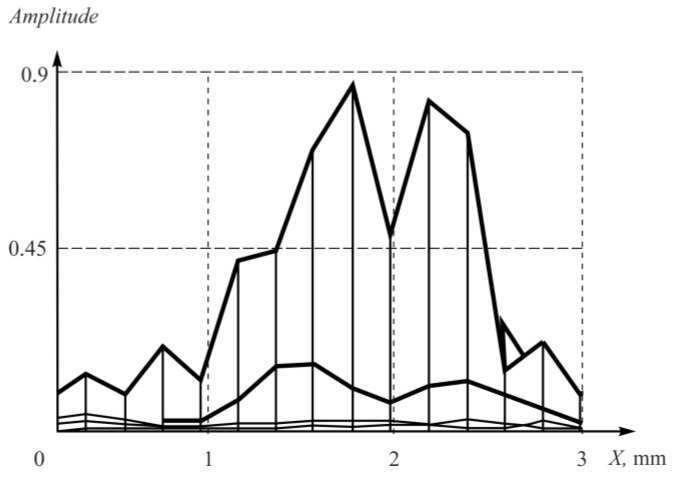
The second maximum appearance caused by the shift.

As seen in Figure 13a, the Strehl ratio possessed nonlinear character. It decreased gradually from 1.00 to 0.74 within the range of the coordinates shift of the flexible acoustic array from 0 to 0.33λ. Within the coordinates shift from 0.33λ to 0.50λ, the Strehl ratio changed insignificantly; while with further increase in the coordinates shift up to 0.66λ, the Strehl ratio decreased to 0.63. Within the range from 0.66λ to 0.83λ, the Strehl ratio increases to 0.9. A further increase in the shift value of more than 0.83λ lead to a decrease in the Strehl ratio. Such aberrations of the Strehl ratio were related to changes of A-scan phase caused the coordinates shift. The coordinate shift equal to 0.50λ lead to the changes in the phase of A-scans to 180 degrees, which caused the maximum reduction in the central maximum amplitude of the point reflector. The phase of A-scans will change to 360 degrees when the shift is about 1λ and it will increase of the central maximum amplitude of the point reflector. Along with the increasing of the central maximum amplitude, the second maximum appeared on the reconstructed image (Figure 14).

The discussed results were obtained for the point reflector placed on the distance of 24 mm from the central element of the convex array of 64 elements. Similar results were obtained for the distances of 35, 42 and 50 mm. The deviation of the Strehl ratio was below 6%.

The experimental studies of the Strehl ratio for the concave acoustic array were practically identical to the results obtained or the convex array. The deviation of obtained values did not exceed 3%. The appearance of the second maximum was observed for the coordinate shift of the acoustic array elements for 1.1λ.

The Strehl ratio equal to 0.8 was obtained when the coordinates shift of the flexible acoustic array elements within the range ±0.2λ.

## 5. Conclusions

In the article, we discussed the focusing quality of TFM tomogram when the coordinates of the acoustic array elements were incorrectly calculated. In order to analyze this case, the simulation of acoustic inspection was performed for the point reflector. The Strehl ratio was chosen as the criterion for focusing quality evaluation in order to reach the goal of the study and define the quantitative limits of the acceptable deviation of the elements’ coordinates values. The tomogram was reconstructed both using the precise coordinates of the acoustic array elements and the shifted ones. Moreover, even and odd elements of the acoustic array were shifted in opposite directions, which corresponded to the worst possible error. The amplitude of the central maximum of the reflector was measured for each described case. These values were used to calculate the Strehl ratio. The simulation results showed that, according to the Strehl ratio, the error in determination of the coordinates should not exceed 18% of the wavelength. 

During experimental validation of the simulation’s results, the point reflector was located in the RoI and a complete set of A-scans was obtained by DOPPLER flexible acoustic array. Applying experimental set of A-scans, the tomogram was reconstructed with the precise coordinates and with the shifted one. To determine the Strehl ratio, the same approach was used as in the simulation procedure. Experimental validation showed that the acceptable shift of the array element coordinates was 0.2λ. In addition, the conducted studies showed that when the coordinates of the acoustic array elements were shifted by λ, the second maximum would appear, which would lead to a false conclusion about the presence of two reflectors in the RoI. The obtained results show the necessity of determining the coordinates of the elements of the flexible acoustic array with a deviation of less than 0.18λ in order to obtain a focused tomogram of the RoI and exclude the appearance of artifacts.

## Figures and Tables

**Table 1:** The value of the acceptable shift for the elements of the concave array with the curvature radius 60 mm.

The Shift, mm	The Distance between the Array and the Point Reflector, mm					
	5	10	15	24	35	50
0	1.000	1.000	1.000	1.000	1.000	1.000
0.05	0.988	0.993	0.994	0.991	0.992	0.991
0.10	0.948	0.953	0.961	0.957	0.961	0.962
0.15	0.859	0.863	0.861	0.858	0.857	0.862
0.20	0.750	0.737	0.739	0.738	0.729	0.728
0.25	0.610	0.598	0.598	0.595	0.587	0.593
0.30	0.471	0.474	0.469	0.473	0.468	0.469
0.35	0.351	0.346	0.339	0.352	0.347	0.338
0.40	0.252	0.245	0.238	0.251	0.245	0.239
0.45	0.173	0.171	0.175	0.172	0.171	0.174
0.50	0.154	0.156	0.151	0.153	0.155	0.152
0.55	0.181	0.177	0.183	0.186	0.188	0.182
0.60	0.213	0.211	0.217	0.216	0.214	0.216
0.65	0.277	0.280	0.283	0.270	0.282	0.286
0.70	0.350	0.362	0.364	0.351	0.365	0.365
0.75	0.429	0.434	0.437	0.419	0.439	0.439
0.80	0.487	0.497	0.502	0.483	0.499	0.500
0.85	0.563	0.580	0.587	0.561	0.585	0.588
0.90	0.639	0.644	0.648	0.637	0.649	0.651
0.95	0.701	0.701	0.703	0.695	0.702	0.705
1.00	0.739	0.736	0.750	0.724	0.732	0.748
1.10	0.704	0.709	0.706	0.707	0.709	0.705
1.20	0.632	0.635	0.640	0.634	0.632	0.639
1.30	0.599	0.593	0.596	0.597	0.595	0.597
1.40	0.529	0.528	0.537	0.532	0.534	0.534
1.50	0.459	0.462	0.460	0.463	0.461	0.465

**Table 2:** The point reflector amplitude values.

The Shift, mm	The Shift, in Wavelengths	The Amplitude of the Central Maximum	The Strehl Ratio
0	0	10.43	1.000000
0.05	0.166667	8.61	0.825503
0.10	0.333333	7.72	0.740173
0.15	0.500000	7.70	0.738255
0.20	0.666667	6.58	0.630872
0.25	0.833333	9.40	0.901246
0.30	1.000000	8.68	0.832215
0.35	1.166667	6.90	0.661553
0.40	1.333333	6.00	0.575264

## Data Availability

The data that support the findings of this study are available from the corresponding author upon reasonable request.

## References

[1] (2017). Introduction to Phased Array Ultrasonic Technology Applications.

[2] Huang J., Zhou S.-y., Zhou S.-g., Que P.-w. Characteristic Research on Focused Acoustic Field of Linear Phased Array Transducer. Proceedings of the 9th International Conference on Electronic Measurement & Instruments.

[3] Berke M., Bechler J. Ultrasonic Imaging in Automatic and Manual Testing. Proceedings of the 9th European Conference on NDT.

[4] Pain D., Drinkwater B.W. (2013). Detection of Fibre Waviness Using Ultrasonic Array Scattering Data. J. Nondestruct. Eval..

[5] Hunter A.J., Drinkwater B.W., Wilcox P.D. (2008). The wavenumber algorithm for full-matrix imaging using an ultrasonic array. IEEE Trans. Ultrason. Ferroelectr. Freq. Control..

[6] Orlakan O., Ergun S., Johnson J.A., Karaman M., Demiciri U., Kaviani K., Lee T.H., Khuri-Yacub B.T. (2002). Capacitive micromachined ultrasonic transducers: Next-generation arrays for acoustic imaging?. IEEE Trans. Ultrason. Ferroelectr. Freq. Control..

[7] Zhang J., Drinkwater B.W., Wilcox P.D., Hunter A.J. (2010). Defect detection using ultrasonic arrays: The multi-mode total focusing method. NDT E Int..

[8] Bolotina I., Dyakina M., Kröning M., Mohr F., Reddy K.M., Soldatov A., Zhantlessov Y. (2013). Ultrasonic arrays for quantitative nondestructive testing an engineering approach. Russ. J. Nondestruct. Test..

[9] Wilcox P.D., Holmes C., Drinkwater B.W. Enhanced Defect Detection and Characterisation by Signal Processing of Ultrasonic Array Data. Proceedings of the 9th European Conference on NDT.

[10] Velichko A., Wilcox P.D. (2011). Defect characterization using two-dimensional arrays. AIP Conf. Proc..

[11] Velichko A., Wilcox P.D. (2010). Strategies for Ultrasound Imaging Using Two-Dimensional Arrays. AIP Conf. Proc..

[12] Bancroft J. (2002). A visual relationship between Kirchhoff migration and seismic inversion. CREWES Res. Rep..

[13] Bannouf S., Robert S., Casula O., Prada C. (2012). Evaluation of multi-element methods applied to complex geometries. AIP Conf. Proc..

[14] Toullelan G., Casula O., Abittan E., Dumas P. (2008). Application of a 3D smart flexible phased-array to piping inspection. AIP Conf. Proc..

[15] Lane C.J.L. (2014). The inspection of curved components using flexible ultrasonic arrays and shape sensing fibres. Case Stud. Nondestruct. Test. Eval..

[16] Casula O., Toullelan G., Roy O., Dumas P. Ultrasonic Nondestructive Testing of Complex components with Flexible Phasedarray Transducers. Proceedings of the 10th European Conference on Non-Destructive Testing.

[17] Mahaut S., Porré J., Calmon P., Chatillon S., Roy O. Simulation and Application of Phased Array Techniques to NDT of Complex Structures. Proceedings of the World Congress on Ultrasonics.

[18] Robert S., Calmon P., Calvo M., Jeune L.L., Iakovleva E. (2015). Surface Estimation Methods with Phased-Arrays for Adaptive Ultrasonic Imaging in Complex Components. AIP Conf. Proc..

[19] Jeune L.L., Robert S., Dumas P., Membreo A., Prada C. (2015). Adaptive Ultrasonic Imaging with the Total Focusing Method for Inspection of Complex Components Immersed in Water. AIP Conf. Proc..

[20] Changliang S., Yimin L., Rutao G., HongYan Y. (2020). Comprehensive Nondestructive Evaluation Technology for Safety and Reliability of Engine Crankshaft. Mater. Trans..

[21] Aliakbari K., Nejad R.M., Toroq S.K.P., Macek W., Branco R. (2022). Assessment of unusual failure in crankshaft of heavy-duty truck engine. Eng. Fail. Anal..

[22] Tian L., Ding N., Liu L., Xu N., Guo W., Wu X., Xu H., Wu C.-M.L. (2023). Fracture failure of the multi-throw crankshaft in a sport utility vehicle. Eng. Fail. Anal..

[23] Nejad R.M., Liu Z., Ma W., Berto F. (2021). Reliability analysis of fatigue crack growth for rail steel under variable amplitude service loading conditions and wear. Int. J. Fatigue.

[24] Wang H., Yang S., Han L., Fan H., Jiang Q. (2020). Failure analysis of crankshaft of fracturing pump. Eng. Fail. Anal..

[25] Brown R.H., Dobson J., Pierce S.G., Dutton B., Collison I. (2017). Quantifying performance of ultrasonic immersion inspection using phased arrays for curvilinear disc forgings. AIP Conf. Proc..

[26] Kwon H., Joh C., Chin W.J. (2021). Pulse Peak Delay-Total Focusing Method for Ultrasonic Tomography on Concrete Structure. Appl. Sci..

[27] Kachanov V.K., Sokolov I.V., Kontsov R.V., Timofeev D.V. (2019). Using “Focusing to a Point” Algorithm for Reference-Free Measurement of the Speed of Ultrasound In Tomography of Concrete Engineering Structures. Russ. J. Nondestruct. Test..

[28] Filho J.F.M.R., Bélanger P. (2021). Probe Standoff Optimization Method for Phased Array Ultrasonic TFM Imaging of Curved Parts. Sensors.

[29] Guan S., Wang X., Hua L., Zeng Y. (2022). Ultrasonic phased array inspection of aeroengine casing ring forgings using adaptive filtering and angle gain compensation algorithm. Appl. Acoust..

[30] Niu K., Tian C. (2022). Zernike polynomials and their applications. J. Opt..

[31] Bos A.v.d. (1999). Rayleigh wave-front criterion: Comment. J. Opt. Soc. Am. A.

[32] Sheppard C.J.R. (2014). Maréchal condition and the effect of aberrations on Strehl intensity. Opt. Lett..

[33] Mahajan V.N. (1983). Strehl ratio for primary aberrations in terms of their aberration variance. J. Opt. Soc. Am..

[34] Doppler Product Manual. https://melcondt.com/wp-content/uploads/2019/10/Doppler-Brochure-2019.pdf.

[35] Lozak A., Boller C., Bulavinov A., Pinchuk R., Kurz J., Sednev D. Phase Statistics and Spectral Analysis of Ultrasonic Signals for CFRP Component Assessment. Proceedings of the 7th European Workshop on Structural Health Monitoring.

